# The Valerianate of Zinc*Lancet, from *Bulletino delle Scienze Medicale*.

**Published:** 1844-10

**Authors:** 


### SELECTIONS

FROM

### AMERICAN AND FOREIGN JOURNALS.

---

*The Valerianate of Zinc.** By MM. Muratori and Cerulli.—This new therapeutical agent is frequently used in Italy, in the treatment of nervous diseases. Louis Bonaparte, it appears, was the person whose researches popularised the new salt, the efficacy of which might have been anticipated from the known properties of the substances by which it is formed. M. Cerulli gives it in doses of one grain and a half daily, in the form of pills, which he administers at the time of the paroxysm. He has thus cured several cases of orbital neuralgia; one in thirty, one in forty, and a third in fifty days. The mode of preparation is as follows:—The valerianic acid is introduced into a retort, and the hydrated protoxide of zinc is slowly added unto saturation. Heat favors the combination. The saline solution is poured into a porcelain capsule, and a small quantity of protoxide of zinc is added, in order to salify all the valerianic acid. The dissolution is then concentrated by evaporation; a white crust of valerianate of zinc forms on the surface. It is taken off as it forms, dried, and preserved in a bottle. M. Muratori has also obtained this salt by the double decomposition of sulphate of zinc and of valerianate of lime. By filtering, the valerianate of zinc, which is soluble, is separated from the sulphate of lime, which is insoluble. The dissolution is then concentrated.

---

*Antagonism of Pulmonary Phthisis and of Intermittent Fever.*—At the scientific congress of Lucca this subject was warmly discussed, without any satisfactory result having been

---

* Lancet, from *Bulletino delle Scienze Medicale.*

